# *Killer Immunoglobulin-like Receptor (KIR)* and *HLA* Genotypes Affect the Outcome of Allogeneic Kidney Transplantation

**DOI:** 10.1371/journal.pone.0044718

**Published:** 2012-09-13

**Authors:** Izabela Nowak, Maria Magott-Procelewska, Agnieszka Kowal, Maciej Miazga, Marta Wagner, Wanda Niepiekło-Miniewska, Małgorzata Kamińska, Andrzej Wiśniewski, Edyta Majorczyk, Marian Klinger, Wioleta Łuszczek, Andrzej Pawlik, Rafał Płoski, Ewa Barcz, David Senitzer, Piotr Kuśnierczyk

**Affiliations:** 1 Laboratory of Immunogenetics and Tissue Immunology, Department of Clinical Immunology, Ludwik Hirszfeld Institute of Immunology and Experimental Therapy, Polish Academy of Sciences, Wrocław, Poland; 2 Department and Clinic of Nephrology and Transplant Medicine, Faculty of Medicine, Medical University of Wroclaw, Wrocław, Poland; 3 Health Care Center at the Ludwik Hirszfeld Institute of Immunology and Experimental Therapy, Wrocław, Poland; 4 Department of Pharmacokinetics and Therapeutic Drug Monitoring, Pomeranian Medical University, Szczecin, Poland; 5 Department of Medical Genetics, Centre of Biostructure Research, Medical University of Warsaw, Warsaw, Poland; 6 1st Chair and Clinic of Obstetrics and Gynecology, Medical University of Warsaw, Warsaw, Poland; 7 City of Hope Comprehensive Cancer Center, Duarte, California, United States of America; Hospital Infantil Universitario Niño Jesús, Spain

## Abstract

**Background:**

Recipient NK cells may detect the lack of recipient's (i.e., self) HLA antigens on donor renal tissue by means of their killer cell immunoglobulin-like receptors (KIRs). *KIR* genes are differently distributed in individuals, possibly contributing to differences in response to allogeneic graft.

**Methodology/Principal Findings:**

We compared frequencies of 10 *KIR* genes by PCR-SSP in 93 kidney graft recipients rejecting allogeneic renal transplants with those in 190 recipients accepting grafts and 690 healthy control individuals. HLA matching results were drawn from medical records. We observed associations of both a full-length *KIR2DS4* gene and its variant with 22-bp deletion with kidney graft rejection. This effect was modulated by the *HLA-B,-DR* matching, particularly in recipients who did not have glomerulonephritis but had both forms of *KIR2DS4* gene. In contrast, in recipients with glomerulonephritis, *HLA* compatibility seemed to be much less important for graft rejection than the presence of *KIR2DS4* gene. Simultaneous presence of both *KIR2DS4* variants strongly increased the probability of rejection. Interestingly, *KIR2DS5* seemed to protect the graft in the presence of *KIR2DS4fl* but in the absence of *KIR2DS4del*.

**Conclusions/Significance:**

Our results suggest a protective role of *KIR2DS5* in graft rejection and an association of *KIR2DS4* with kidney rejection, particularly in recipients with glomerulonephritis.

## Introduction

Acute or chronic rejection of solid organ grafts such as kidney is mediated by alloreactive T lymphocytes recognizing major (HLA) and minor histocompatibility antigens by means of antigen-specific T cell receptors (TCR) [1], [2]. However, a contribution of natural killer (NK) cells has also been postulated. Thus, infiltration of renal allografts by NK cells [3]–[5], increased numbers of NK cells in peripheral blood of patients acutely rejecting kidney graft [6], and increased cytotoxicity of recipient NK cells against donor peripheral blood cells in vitro were described [7].

NK cells recognize the presence of HLA class I (HLA I) molecules on the surface of potential target cells using several types of the receptors, among them polymorphic killer cell immunoglobulin-like receptors (KIRs). Normal cells of an individual are spared by his or her NK cells because they express normal level of cell surface HLA I molecules detected by NK cell inhibitory receptors. However, virus-infected or neoplastic cells tend to lose HLA I expression, and may be eliminated by NK cells [8].

Due to HLA and KIR polymorphism, in some combinations of the graft donor and recipient, recipient NK cell's inhibitory KIRs may not bind HLA I molecules present on donor cells, leading to NK cell alloreactivity against the transplanted organ, similarly to the reaction in opposite direction in hematopoietic cell transplantation [9]–[11]. In addition, KIRs are expressed also on some T lymphocytes, particularly on special subpopulation of CD4+CD28− cytotoxic T cells involved in autoimmune vasculitis [12]–[14], potentially influencing their activity in graft rejection.

Human KIRs are encoded by genes located in the chromosomal region 19q14. KIR genetics is characterized by both allelic (up to more than 50 alleles for some *KIRs*) and haplotypic (i.e., different numbers of inhibitory and activating genes on individual chromosomes) polymorphism [8], [15]. As a result, above 97% of unrelated persons differ by their *KIR* genotype [16]–[17]. Two categories of *KIR* haplotypes were described: A-type haplotypes containing mostly inhibitory *KIRs*, and only *KIR2DS4* and *KIR2DL4* as activating ones, and B-type haplotypes, characterized by one or more of other activating *KIRs* in addition to inhibitory ones. For this reason, people may differ substantially in their NK and T cell responses, depending on *KIR* genotype. We have recently published results showing a contribution of *KIR2DS5* gene to a tolerance of kidney graft as well as to other clinical situations [18]. Here, we focused on kidney graft rejection and compared frequencies of 10 *KIR* genes in recipients rejecting the allogeneic renal transplant with those in recipients accepting such a graft. Our study is the first report on different HLA and KIR genetic associations of kidney graft acute rejection in recipients whose pre-transplant renal failure resulted from glomerulonephritis versus those whose renal failure was a result of other disease.

## Materials and Methods

### Kidney graft recipients and controls

**Figure 1:**
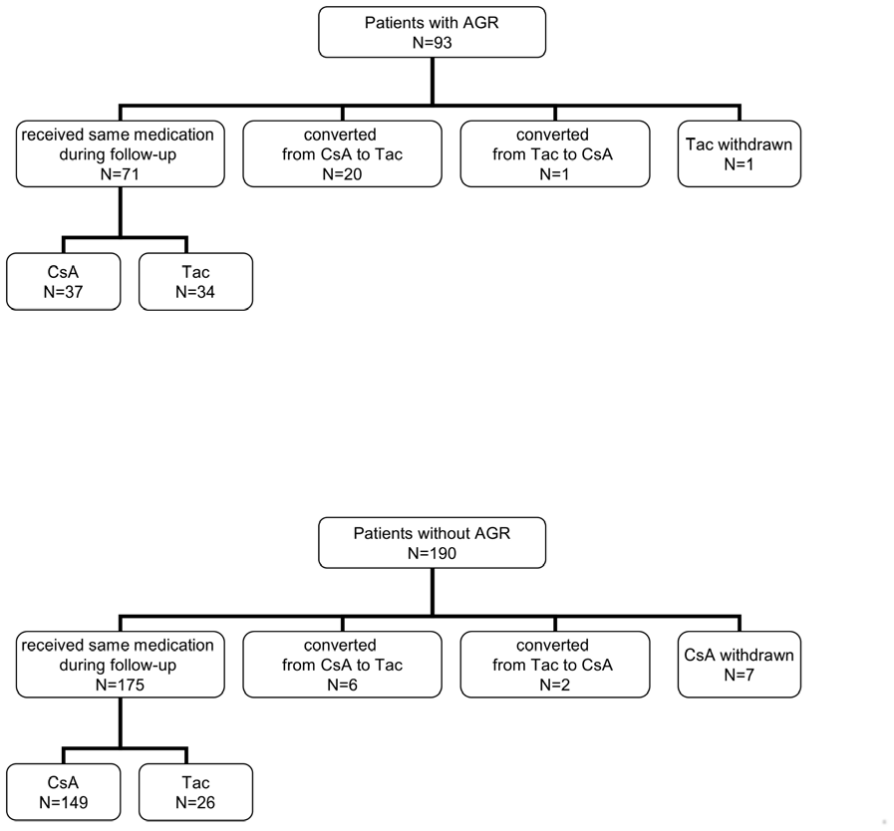
Patient disposition according to initial calcineurine inhibitor use. AGR, acute graft rejection; CsA, cyclosporine A; Tac, Tacrolimus.

**Figure 2:**
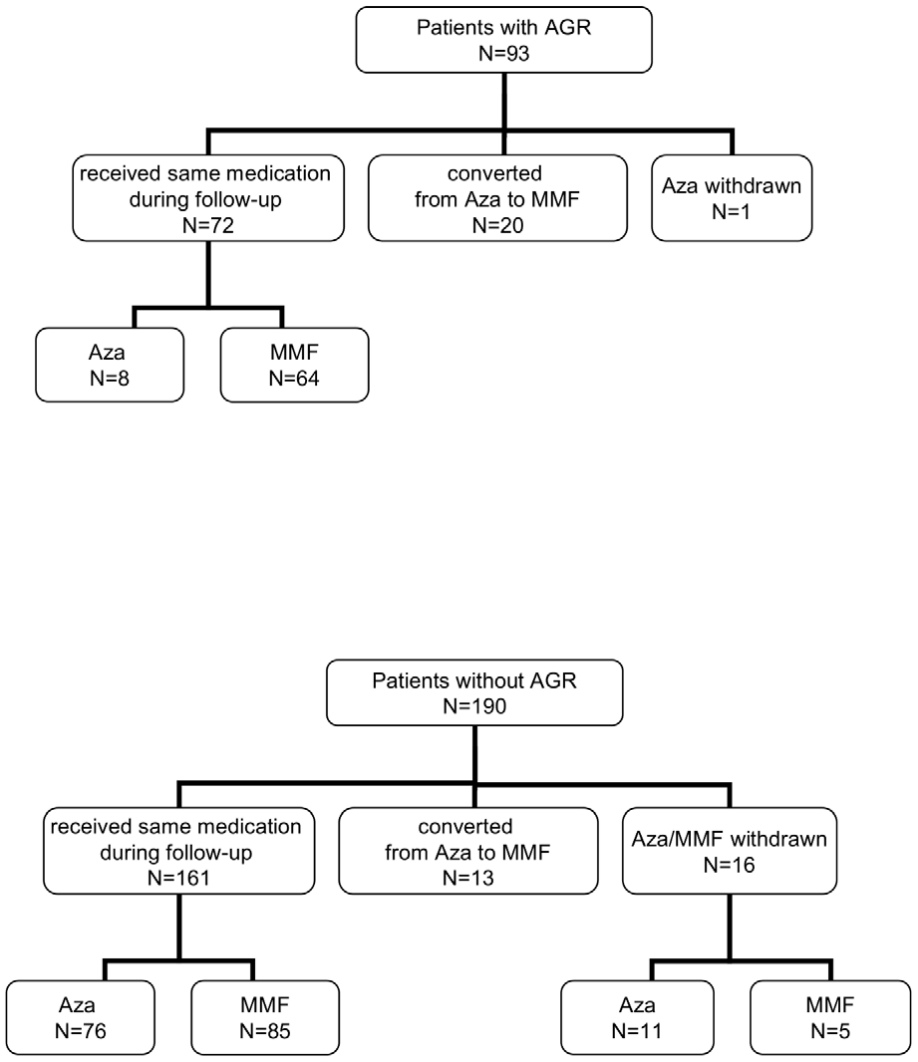
Patient disposition according to purine metabolism inhibitor use. AGR, acute graft rejection; Aza, azathioprine; MMF, mycophenolate mofetil.

All individuals, including kidney graft recipients, donors, and healthy controls, were Polish Caucasians. Two hundred eighty-three kidney patients (clinical data presented in **Table 1**) underwent first transplantation and received deceased donor kidney between 1989 and 2008 (166 patients after 2000). All patients were treated with triple-therapy (**Figure 1**) as initial immunosuppression that incorporated cyclosporine (n = 219) or tacrolimus (n = 64, beginning in 2000) in combination with azathioprine (n = 129) or mycofenolate mofetil (n = 154, since 1998) (**Figure 2**) and steroids. No induction with antibodies was used. During the follow up (mean time was 7 years) there were 246 (87%) patients who were treated with the same calcineurin inhibitor. Among 29 patients who changed the type of calcineurin inhibitor, 20 patients were converted from CsA to tacrolimus after an episode of rejection treated with methylprednisolone. Calcineurin inhibitor was withdrawn in 8 individuals. There were 233 patients who received the same type of purine metabolism inhibitor during follow up: azathioprine (n = 84) and mycofenolate mofetil (n = 149). Azathioprine was replaced by mycofenolate mofetil in 33 patients (in 20 patients after an episode of acute rejection) or stopped in 12 patients. The frequency of a change in treatment regimen was almost 2-fold higher in patients who suffered a rejection (refers to 38% and 21% of patients with and without rejection, respectively, p = 0.0063).

**Table 1:** Clinical characteristics of patients.

		Patients with AGR	Patients without AGR	P	OR	95%CI
		N = 93	N = 190			
**Age**	Mean +/− SD	43.65±11.31	43.36±11.46	0.8		
	min-max	15–67	16–72			
**Sex**	Females/Males	32/61	92/98	0.03	0.56	0.33–0.93
	% of Females	34.4%	48.4%			
		N (%)	N (%)			
**Cause**	Glomerulonephritis	58 (62.4)	74 (39.0)	0.0002	2.60	1.56–4.33
**of**	Interstitial nephritis	10 (10.7)	27 (14.2)	0.46	0.73	0.34–1.58
**renal**	Cystic kidney	10 (10.7)	24 (12.6)	0.7	0.83	0.38–1.82
**failure**	Hypertensive nephropathy	3 (3.2)	13 (6.8)	0.28	0.45	0.13–1.63
	Diabetic nephropathy	5 (5.4)	7 (3.7)	0.54	1.49	0.46–4.81
	Other	7 (7.6)	45 (23.7)	0.0009	0.26	0.11–0.61

93 recipients exhibited symptoms of acute graft rejection based on clinical criteria (an increase in serum creatinine level of at least 20% above the baseline measurements not attributable to another cause) confirmed by histopathological examination according to Banff criteria [19]. Apart from three patients, all had a biopsy-confirmed acute rejection episode. Remaining 190 recipients experienced stable graft function during long-term follow-up. 31 (33%) patients who suffered acute rejection subsequently lost their grafts in comparison to 22 (12%) patients without an episode of rejection. During the follow-up, 5 out of 93 (5.4%) patients with AGR and 13 out of 190 (6.8%) patients without AGR died for different reasons.

Six hundred and ninety unrelated healthy volunteers, constituting a basic control group in KIR studies performed in our laboratory, were recruited in the years 2001–2008 by the Regional Center of Blood Transfusion, Wroc^3^aw, as well as by clinics of the Wroc^3^aw Medical University, the Medical University of Warsaw, and the Pomeranian Medical University, Szczecin.

The same cohorts of patients and controls have already been used to describe a protective effect of *KIR2DS5* gene on kidney graft rejection and some other clinical situations [18].

The Bioethics Committee of the Wroc^3^aw Medical University specifically approved this study. Signed written informed consent was given by all participants.

### DNA isolation and KIR typing

DNA was isolated from venal blood as described [20], [21]. The presence or absence of *KIR* genes was detected by either individual [20]–[22] or multiplex [23] polymerase chain reactions (PCR) which, when tested on the same samples, gave virtually identical results. Our *KIR* typing has been validated three times per year by the International KIR Exchange program organized by the Immunogenetics Center of the University of California at Los Angeles.

*HLA-A*, *-B*, and *-DR* typing of donors and recipients has been routinely done before transplantation either in Non-Public Tissue Typing Facility at our Institute or in other transplant centers in Poland, and it was drawn from the clinical histories of the patients. Tissue samples were available for only 42 donors, therefore their *HLA-C* typing was possible only in these instances, had statistically insufficient power, and therefore its results are not presented here. Recipient *HLA-C* variants encoding C1 and C2 epitopes were described and discussed earlier [18].

### Statistical analysis

General linear model (GLM) with binomial errors was used to investigate relationship between clinical and genetic variables and probability of rejecting the transplanted kidney (**Table 2**). Frequencies of *KIR* genes in recipients and *HLA* matching between donors and recipients were explanatory variables. Clinical characteristics (age, sex, creatinine, course of transplantation and time of observation) was concomitant variables. Akaike's information criterion (AIC) was used as a measure of fit of models. *Bootstrap* approach was employed to estimate model's coefficients and 95% confidence intervals. Chi-squared test with Yates' continuity correction was used to test hypothesis that rejection and type of genotype were independent. To test differences in *KIR*'s distribution among patients and control, a group permutation test was employed. This procedure was based on Mahalanobis distance (D_M_) between two groups and test performed in 10 000 permutations. *Odds ratio* (OR) was computed as a measure of effect size. Probability that graft is not rejected at a given time, S(t), was computed according to the Kaplan-Meier method, comparing *KIR* genotypes. Haplotype frequencies (HFs) among two *KIRs*: *2DS4* (full-length or deletion variant) and *2DS5* were estimated with *maximum likelihood* function [24].

**Table 2:** Variables significantly associated with probability of graft rejection.

Variable	OR	95% CI		P
HLA-B+DR match	0.46	0.30	0.70	0.0003
KIR2DS4fl	2.02	1.05	3.90	0.03
KIR2DS4del	2.61	1.19	5.75	0.02
HLA-B+DR match×GN	2.01	1.46	2.76	0.0000

Abbreviations: CI, confidence interval; GN, glomerulonephritis; HLA, human leukocyte antigen; KIR, killer immunoglobulin-like receptor; *KIR2DS4fl*, *KIR2DS4* full length gene; *KIR2DS4del*, *KIR2DS4* 22-base pair deletion variant of the *KIR2DS4* gene; OR, odds ratio.

Measures for the estimation of linkage disequilibrium (LD) were the correlation of two alleles frequencies, *r*, global squared correlation between two loci, R^2^ and Kullback-Leibler divergency two loci from LE [24], [25]. For two loci *2DS4* and *2DS5*, *r* and R^2^ obtained as: 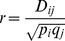, where 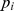 and 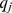 are the population allele frequencies of the 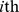 allele on locus 2DS4 and the 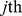 allele on locus *2DS5*, 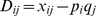, and 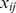 is the frequency of the haplotype with alleles 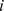 and 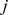 on loci *2DS4* and *2DS5*, respectively. 
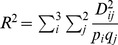
. Kullback-Leibler divergence [26], [27], 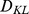, is a measure of distance between the observed haplotype distribution and the expected distribution assuming LE: 
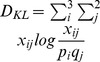
. Chi-square statistic was calculated to test that all of the 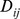
's between 2DS4 and 2DS5 are zeros: 
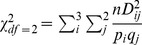
. Likelihood ratio statistic, LRS, was used to test for differences in haplotype frequencies between rejectors, non-rejectors and controls.


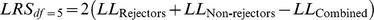
, where log likelihoods were produced based on haplotype frequencies and 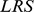 is approximately a χ^2^. Results were regarded as statistically significant at p<0.05. All data were analyzed using R version 2.2.1.

## Results

### *HLA* and *KIR2DS4* gene effects on acute kidney graft rejection

**Figure 3:**
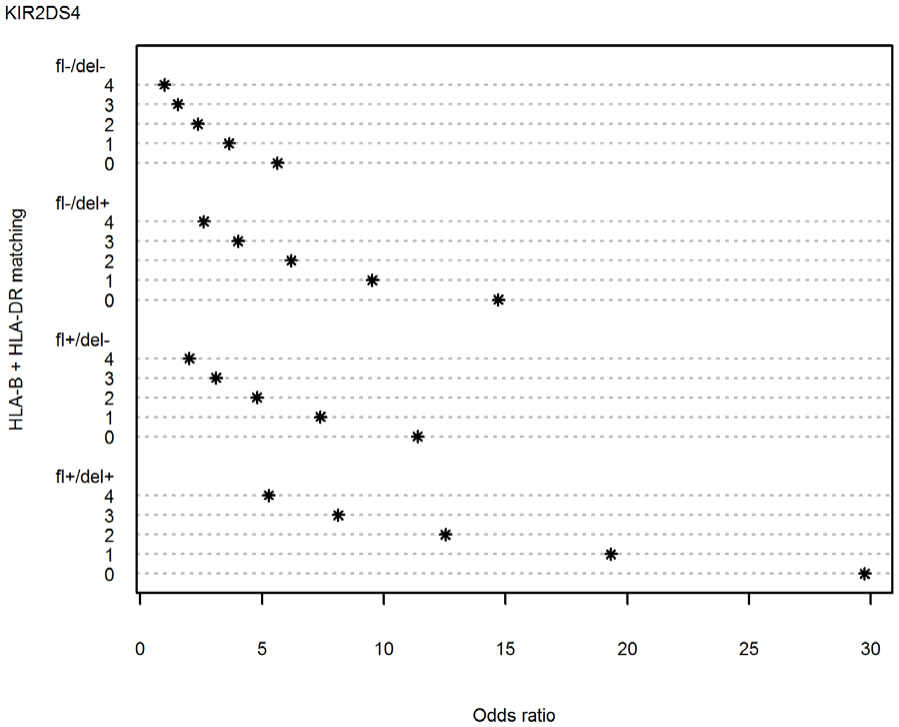
Dependence of odds ratio for kidney graft rejection on *HLA-B,-DR* matching, *KIR2DS4* full length (*KIR2DS4fl*) and/or *KIR2DS4* deletion variant (*KIR2DS4del*) gene presence. For odds ratio calculations, the recipient group with complete (n = 4) *HLA-B,-DR* match with the donor and a lack of any *KIR2DS4* variant (*KIR2DS4fl* and *KIR2DS4del* negative: fl-/del-) was taken as 1.

Frequencies of *KIR* genes were not different between patients and controls (**Table 3**). However, there were some differences between patients with acute graft rejection (AGR) (determined by Banff criteria) and patients without AGR. First, the frequency of the *KIR2DS5* gene in patients with AGR was two times lower than in control individuals (p = 0.0056). This protective effect of *KIR2DS5* gene on kidney graft rejection has already been published recently on the same cohorts of patients and controls [18]. Multivariate analysis indicated significant protective effect of *HLA-B,-DR* matching (**Table 2**) but HLA-A did not affect graft fate (data not shown). We also observed that the presence of both *KIR2DS4* full-length (*KIR2DS4fl*) and 22-base pair deletion variant (*KIR2DS4del)* gene was increasing a probability of rejection at least twofold (**Table 2**). This effect was amplified to a great extent by the *HLA-B,-DR* mismatching (matching = 0, **Figure 3**).

**Table 3:** *KIR* gene frequencies in controls and patients.

							KIR					
Group		2DL1	2DL2	2DL3	2DS1	2DS2	2DS3	2DS4fl	2DS4del	2DS5	3DL1	3DS1
Patients	Present	268	142	257	105	143	81	82	236	65	256	93
N = 283	%	94.70	50.18	90.81	37.10	50.53	28.62	28.97	83.39	22.97	90.46	32.86
Control	Present	665	374	623	299	369	214	194	561	205	642	264
N = 690	%	96.38	54.20	90.29	43.33	53.48	31.01	28.12	81.30	29.71	93.04	38.26
	*p*	0.3	0.3	0.9	0.07	0.4	0.5	0.8	0.5	0.03	0.2	0.1
	OR	0.67	0.85	1.06	0.77	0.89	0.89	1.04	1.55	0.71	0.71	0.79
	95%CI	0.35–1.29	0.65–1.12	0.66–1.71	0.58–1.03	0.67–1.17	0.66–1.21	0.77–1.42	0.80–1.67	0.51–0.97	0.43–1.16	0.59–1.06

Abbreviations: CI, confidence interval; KIR, killer immunoglobulin-like receptor; *KIR2DS4fl*, *KIR2DS4* full length gene; *KIR2DS4del*, *KIR2DS4* 22-base pair deletion variant of the *KIR2DS4* gene; OR, odds ratio.

### Effects of *KIR2DS4* gene variants and *KIR2DS5* gene on the probability of graft rejection

**Figure 4:**
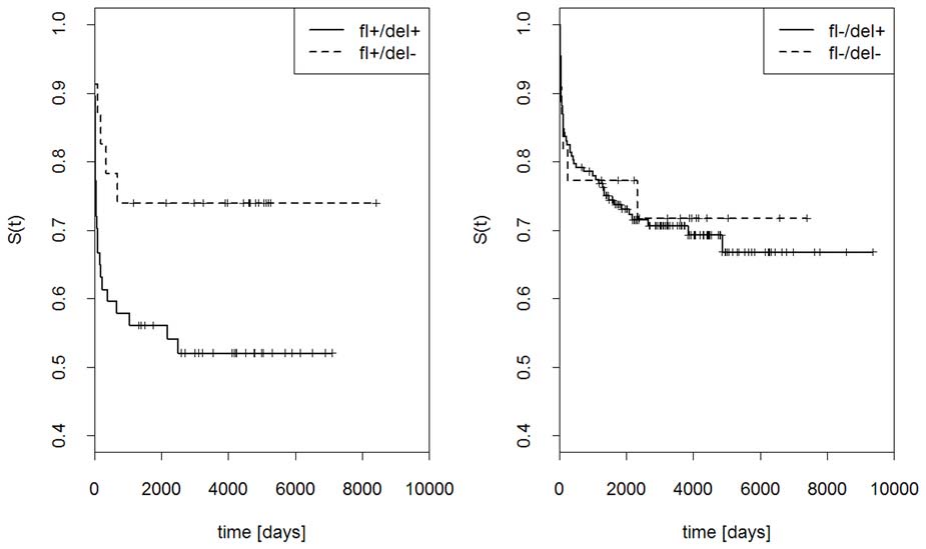
Effects of full-length *KIR2DS4* gene (fl) and its deletion variant (del) on the outcome of renal transplantation. Kaplan Kaplan-Meier estimations of probability that graft is not rejected at a given time, S(t). Left panel: *KIR2DS4fl* present, *KIR2DS4del* present or absent; right panel: *KIR2DS4fl* absent, *KIR2DS4del* present or absent.

**Figure 5:**
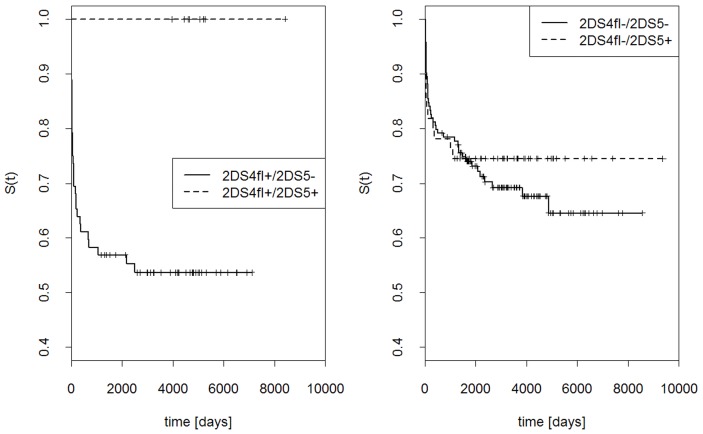
Effects of *KIR2DS5* and full-length (fl) *KIR2DS4* gene on the outcome of renal transplantation. Kaplan Kaplan-Meier estimations as in Figure 4. Left panel: *KIR2DS4fl* present, *KIR2DS5* present or absent; right panel: *KIR2DS4fl* absent, *KIR2DS5* present or absent.

**Figure 6:**
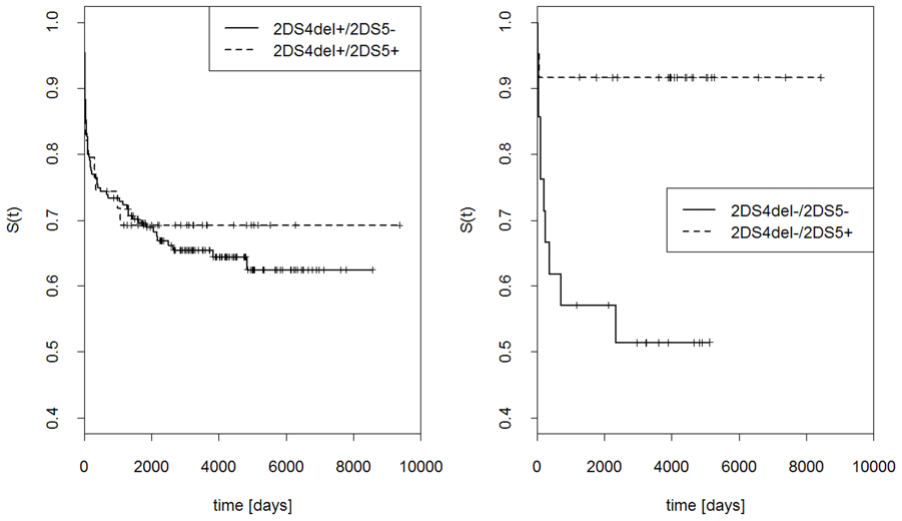
Effects of *KIR2DS5* and *KIR2DS4* deletion variant (*KIR2DS4del*) gene on the outcome of renal transplantation. Kaplan Kaplan-Meier estimations as in Figure 4. Left panel: *KIR2DS4del* present, *KIR2DS5* present or absent; right panel: *KIR2DS4del* absent, *KIR2DS5* present or absent.

Probability that graft was not rejected at a given time, S(t), was computed for the presence or absence of *KIR2DS4fl*, *KIR2DS4del* or both (**Figure 4**). Simultaneous presence of both gene variants strongly increased the probability of graft rejection, whereas the presence of only *KIR2DS4fl*, only *KIR2DS4del*, or none of them gave much lower probability of rejection. Interestingly, the two variants of *KIR2DS4* gene had opposite influence on the effect of *KIR2DS5* gene: *KIR2DS5* seemed to protect the graft stronger in the presence of *KIR2DS4fl* than in its absence (**Figure 5**), but stronger in the absence than presence of *KIR2DS4del* (**Figure 6**).

Combinations of *KIR* genes gave 133 different genotypes present in patients and/or controls (data not shown). These genotypes were divided into AA and BX genotypes, containing two A haplotypes or at least one B haplotype, respectively (for a definition of A and B haplotypes, see Introduction). Two individual AA genotypes were distributed significantly differently between patient subgroups: among patients with the genotype No. 1 acute graft rejection (AGR) was nearly four times less frequent than lack of AGR (21.9% vs 78.1%), whereas in those with the genotype No. 2 the ratio of AGR and non-AGR was 1∶1 (**Table 4**). Interestingly, these two genotypes differed only by the absence of full-length *KIR2DS4* gene in the genotype No. 1 and its presence in the genotype No. 2, and both were devoid of *KIR2DS5* by definition, as AA genotypes. An additional AA genotype containing *KIR2DS4fl* but no *KIR2DS4del* gene was extremely rare (one individual among patients and 8 in controls), therefore it could not play any role in rejection and was omitted from our calculations. Within BX (i.e., non-AA) genotype group, fraction of patients with AGR was nearly two times less frequent than these without AGR. These differences between genotype groups were not accidental (χ^2^ = 6.675, df = 2, p = 0.035).

**Table 4:** Distribution of *KIR* genotype groups in patients with and without acute graft rejection.

	Genotype						KIR						Pts with AGR		Pts without AGR	
No.		2DL1	2DL2	2DL3	2DS1	2DS2	2DS3	2DS4 fl	2DS4 del	2DS5	3DL1	3DS1	N	%	N	%
**1**	**AA/2DS4fl−**	+	−	+	−	−	−	−	+	−	+	−	14	21.9	50	78.1
**2**	**AA/2DS4fl+**	+	−	+	−	−	−	+	+	−	+	−	11	50.0	11	50.0
**3**	**BX**												68	34.5	129	65.5

Differences between frequencies of three genotype groups: χ^2^ = 6.675, df = 2, p = 0.035.

Abbreviations: AA, homozygote for the killer immunoglobulin-like receptor A haplotype (for a definition of A and B haplotypes, see Introduction); AGR, acute graft rejection; BX, a group of the killer immunoglobulin-like receptor genotypes containing at least one B haplotype; as they contain different combinations of KIR genes, the presence or absence of particular genes could not been shown here; KIR, killer immunoglobulin-like receptor; *KIR2DS4fl*, *KIR2DS4* full length gene; *KIR2DS4del*, *KIR2DS4* 22-base pair deletion variant of the *KIR2DS4* gene; Pts, patients.

*KIR2DS4* and *KIR2DS5* are in negative linkage disequilibrium (LD) in all populations tested so far [28]. **Table 5** shows that they are in negative LD also in our population. *KIR2DS4/KIR2DS5* haplotype frequencies for non-rejectors and controls were very similar, and these two groups were combined in the following calculations. Likelihood ratio statistics (LRS) for patients with AGR versus combined group of patients without AGR and controls was high, which suggests that haplotype frequencies in the former were different from those in the latter (p = 0.05). We see, for example, that a haplotype *KIR2DS4−*/*KIR2DS5+* (−*/2DS5*) was three times less frequent in patients with AGR than in other groups. This seems to confirm a protective role of *KIR2DS5* in graft rejection shown above.

**Table 5:** Linkage disequilibrium between *KIR2DS4* gene variants and *KIR2DS5* gene in patients and controls.

Group	Haplotypes	2DS4full/2DS5	2DS4del/2DS5	—/DS25	2DS4full/—	2DS4del/—	—/—
Controls	HFs	0.000	0.006	0.155	0.152	0.558	0.129
	*r*	−0.157	−0.282	0.512	0.069	0.123	−0.224
	R^2^ = 0.436	D_KL_ = 0.306	?^2^ = 300	*df* = 2	p<0.00001		
Patients with AGR	HFs	0.000	0.041	0.048	0.214	0.636	0.061
	*r*	−0.138	−0.079	0.39	0.043	0.025	−0.122
	R^2^ = 0.194	D_KL_ = 0.102	?^2^ = 17.49	*df* = 2	p = 0.00016		
Patients without AGR	HFs	0.005	0.000	0.136	0.131	0.586	0.142
	*r*	−0.138	−0.287	0.489	0.042	0.116	−0.198
	R^2^ = 0.395	D_KL_ = 0.278	?^2^ = 77.06	*df* = 2	p<0.00001		

Abbreviations: AGR, acute graft rejection; HFs, haplotype frequencies; D_KL_, Kullback-Leibler divergence from linkage equilibrium; KIR, killer immunoglobulin-like receptor; *KIR2DS4fl*, *KIR2DS4* full length gene; *KIR2DS4del*, *KIR2DS4* 22-base pair deletion variant of the *KIR2DS4* gene; R^2^, global squared correlation coefficient for two loci; r, correlation coefficient for alleles frequencies. *Likelihood ratio statistics* (LRS): {Pts with AGR} *vs* {Controls+Pts without AGR}; LRS = 11.01; *df* = 5; p = 0.0513.

### Difference between patients with glomerulonephritis and those with other kidney diseases in association of acute graft rejection with *KIR2DS4* and *HLA* genotype

**Figure 7:**
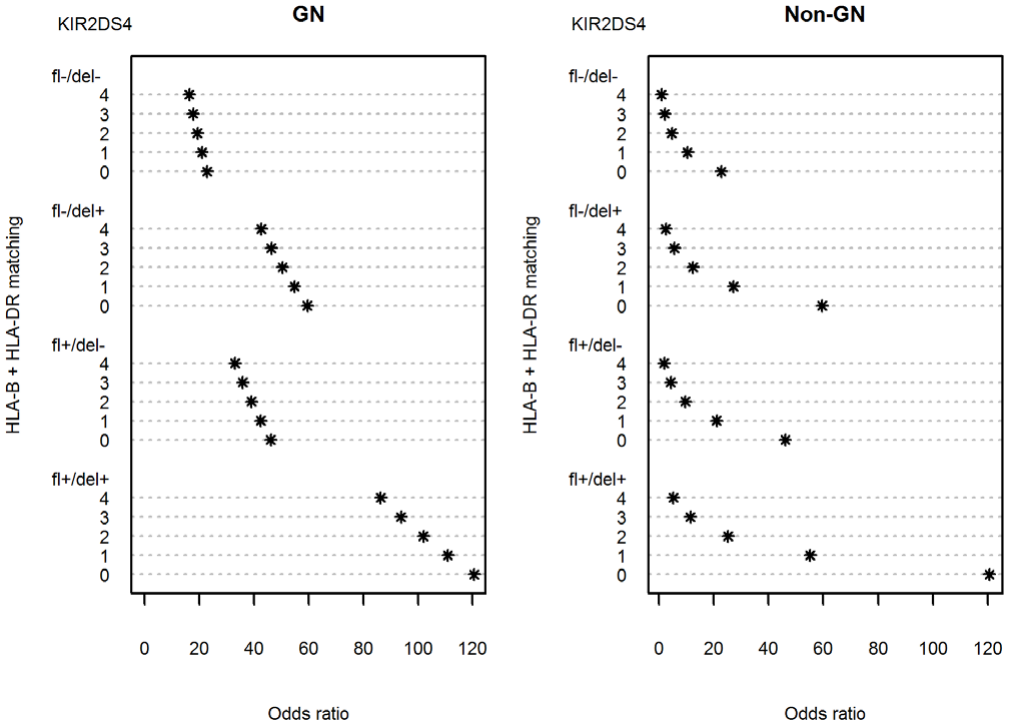
Effect of glomerulonephritis on the dependence of odds ratio for kidney graft rejection on *HLA-B,-DR* matching, *KIR2DS4* full length (*KIR2DS4fl*) and/or *KIR2DS4* deletion variant (*KIR2DS4del*) gene presence. For odds ratio calculations, the non-GN group with complete (n = 4) *HLA-B,-DR* match and lack of any *KIR2DS4* variant (*KIR2DS4fl* and *KIR2DS4del* negative: fl-/del) was taken as 1.

Multivariate analysis revealed also a difference between patients whose end stage renal failure was caused by glomerulonephritis and those with other nephropathies. Namely, in the non-glomerulonephritis group, *HLA-B,-DR* matching seemed to be much more important for acute graft rejection than the presence or absence of *KIR2DS4* gene variants (**Figure 7**, right panel). Thus, in the case of perfect *HLA-B,-DR* matching (matching = 4), the presence or absence of *KIR2DS4fl* and *KIR2DS4del* genes only very weakly influenced graft fate. Recipients of completely *HLA-B,-DR* incompatible grafts (matching = 0) possessing both forms of *KIR2DS4* gene had only 6 times higher chance of kidney rejection than recipients of similarly *HLA-B,-DR*-incompatible grafts negative for *KIR2DS4* (**Figure 7**, right panel).

In contrast, in recipient group with glomerulonephritis, *HLA* incompatibility seemed to be much less important than *KIR2DS4* for graft rejection. For example, completely *HLA*-mismatched *KIR2DS4*-negative recipients had only about 1.4 times higher chance of acute rejection than perfectly *HLA*-matched *KIR2DS4*-negative recipients (**Figure 7**, left panel). On the other hand, the presence of both forms of *KIR2DS4* gene had strong effect on graft rejection in glomerulonephritis group, as even perfect HLA matching did not reduce a chance of rejection below odds ratio of 85 (**Figure 7**, left panel). Individuals from the glomerulonephritis group possessing both variants of *KIR2DS4* and perfect *HLA-B,-DR* matching had about 15 times higher chance of rejection than analogous persons from the non-glomerulonephritis group (see **Figure 7**, both panels).

## Discussion

We compared the distribution of *KIR* genes in patients rejecting and non-rejecting kidney graft as well as in healthy controls. Among individual *KIR* genes, only *KIR2DS4* (both full-length and deletion variants) was remarkably more frequent in patients with AGR than in patients with stable graft function and controls. Moreover, this effect was particularly strong in the absence of *KIR2DS5* gene which exerted opposite effect, i.e., its presence decreased the chance of graft rejection as published already on the same cohorts of patients and controls [18]. It is interesting in this context that KIR2DS4 molecule was expressed on remarkable proportion of CD4+CD28− T cell clones isolated from an acute coronary syndrome patient [29]. Also, dialyzed patients exhibited an increased number of circulating CD4+CD28− T cells [30]. In addition, CMV positivity is universal in our transplant population (data not shown), and the association of CMV infection and the presence of CD4+CD28− cells is well documented (ref.30 and references therein). CD4+CD28− T cells, virtually absent from peripheral blood of healthy individuals but present in acute coronary syndrome and rheumatoid vasculitis [29], multiple sclerosis [14] and, most important here, in end-stage renal disease [30], [31], were found to be resistant to immunoregulation [14] and therefore postulated to play a role in autoimmune diseases and aging [12]. *KIR2DS4fl* encodes an activating receptor which might possibly be involved in stimulation of effector cells (e.g., CD4+CD28− T cells) contributing to transplant rejection, whereas *KIR2DS4del* potentially codes for a soluble molecule [32]. It might be that, in kidney graft recipients, this soluble KIR2DS4 molecule is masking a ligand for membrane-bound KIR2DS4 or another receptor of some regulatory cells (T, NK, or other). The ligand for KIR2DS5 receptor is not known, however it has been observed that the simultaneous presence of *KIR2DS5* gene and *HLA-C*-encoded epitopes for both KIR2DL1 and KIR2DL2/3 receptors significantly decreased leukemia-free survival of hematopoietic stem cell-transplanted patients [33], which could suggest KIR2DS5 interaction with HLA-C. Thus, in the case of renal transplant recipients, soluble KIR2DS4 might block interaction of KIR2DS5 with its ligand which otherwise would protect a graft from rejection. In recipients negative for *KIR2DS4del* gene the presence of *KIR2DS5* seems to favor the acceptance of the graft, particularly in the presence of *KIR2DS4fl* gene. The reason for the latter effect is not clear; either both KIR2DS5 and KIR2DS4fl act in the same direction, e.g. expressed on the same or on two different regulatory cells, or, alternatively, the presence of *KIR2DS4fl* gene, by excluding the presence of its allele, *KIR2DS4del*, from the same chromosome, decreases its frequency in *KIR2DS4fl*-positive patient population.

KIR2DS4 molecule differs from KIR2DS1 and KIR2DS2/3 by weaker interaction with HLA-C and by binding to HLA-A*11 [34]. As mentioned in Material and Methods section, blood, lymphoid tissue or DNA samples of majority of donors were not available for our study, and their *HLA-C* typing was not possible here. However, in a recent study, Hanvesakul and coworkers [35] have not detected any association between **donor** *HLA-C*-encoded KIR ligand and acute rejection of kidney allograft. On the other hand, these authors have reported an effect of **recipient** *HLA-C* on the kidney graft rejection, i.e., a protective effect of C2 on allograft survival. They observed donor-derived NK cells in the allograft at the time of transplantation. These NK cells could stimulate maturation of dendritic cells which would then be capable of indirect stimulation of adaptive immune system for alloreactivity. *In vitro* studies of Hanvesakul et al. [35] have shown that donor-derived, interleukin-15-activated NK cells promoted efficiently the maturation of recipient dendritic cells only when these were C2-negative. They propose that, after kidney transplantation, donor NK cells interacting with recipient C2-positive dendritic cells do not stimulate them for maturation as efficiently as in C2-negative recipients, and this is beneficial for graft survival. In our earlier study, we have not observed such an effect of recipient C2 on graft survival; on the contrary, we have rather seen some, albeit weak and non-significant, association of C2 with kidney rejection [18]. The reason for this discrepancy may lie in numbers: our sample (283 patients) was 2.7 times less numerous than that of the British group (760 individuals). Alternatively, the British and our patients might have differed in a cause of renal failure: we have found a substantial genetic difference between recipients with glomerulonephritis and those with other conditions, whereas Hanvesakul et al. [35] did not show a clinical status of their patients before transplantation.

HLA-A*11 allele (a KIR2DS4 ligand, see above) frequency in the Polish population is 6.239%, as established by Schmidt and colleagues [36]; therefore, its effect on kidney graft survival, if any, could not be strong. Nevertheless, we have noticed that a majority of *HLA-A11*-positive graft-rejecting recipients (4 out of 5) possessed *KIR2DS4fl* gene, whereas only one fifth of A11-positive patients with stable graft function (4 out of 20) typed positive for *KIR2DS4fl* (p = 0.02). However, the numbers were far too small for any conclusion. No difference between distribution of *KIR2DS4del* variant in A11-positive patients with and without AGR was seen (data not shown).

So far, only few publications dealt with *KIR* gene associations with kidney graft rejection. Tran et al. [37] studied an effect of KIR ligand (i.e., C1, C2, and Bw4) matching on graft survival in 1416 recipients of cadaver kidney (both first grafts and regrafts) from all inhabited continents, but did not detect any effect. Similarly negative result was obtained by Kreijveld et al. [38] who tested not only KIR ligands, but also *KIR* genes themselves as well as combinations of both in Dutch population. On the other hand, upon testing a *KIR* polymorphism in *HLA*-identical recipient-donor pairs from U.S.A., Cirocco et al. [39] obtained a result suggesting protective effect of *KIR2DL2* and *KIR2DS2* genes; however, their study was based on extremely low number of individuals (only 12 recipient-donor pairs). Nevertheless, this finding was confirmed by Kunert et al. [40] on 105 graft recipients and 119 controls in Germany. Finally, van Bergen et al. [41] observed an effect of KIR-KIR ligand mismatch between recipient and donor in Dutch population, but only in *HLA-A,-B,-DR*-compatible donor-recipient pairs, i.e., when *HLA*-identical partners differed in their *KIR* gene repertoire, resulting in lack of a ligand in donor for a KIR present in recipient. Notably, the effect of *KIRs* in *HLA*-matched pairs was as strong as that of *HLA-A,-B* mismatch in pairs matched only for *HLA-DR* [41], [42]. In our study, we observed a strong effect of *KIR2DS4* variants in patients with glomerulonephritis, where *HLA-B,-DR* mismatch exerted much weaker influence on the graft fate, whereas in recipients without glomerulonephritis the effect of HLA-mismatch was predominant. The striking finding of this study was the stronger association of *KIR2DS4* polymorphism than *HLA* incompatibility in GN patients. We can speculate that engagement of KIRs in inflammatory pathway activation defined by their polymorphism may represent a common link between autoimune and alloimmune response. The possibility that KIR-ligand interaction may aggravate both the natural history of glomerulonephritis sustaining immune injury leading to end-stage renal disease and influence alloimmune response causing acute rejection cannot be ruled out. In this setting, *KIR2DS4* polymorphism can provoke the immune response as it can modulate autoimmunity. We can hypothesize that GN and non-GN patients may differ in KIR2D receptor expression on NK and T cells, particularly in those rejecting vs. non-rejecting the graft, as it has recently been shown in liver transplantation [43].

In summary, our results suggest that typing of the recipient for *KIR2DS4* and *KIR2DS5* genes may help to predict the outcome of renal transplantation. We show here, for the first time, that the effect of *KIR* genotype on the fate of kidney graft in recipients with glomerulonephritis seems to be stronger than that of *HLA* matching, whereas opposite is true for patients with other causes of end-stage renal disease. The lack of strong association of graft rejection with *HLA-B,-DR* mismatching in recipients with glomerulonephritis could not have been observed in earlier studies done without stratification for the presence or absence of *KIR2DS4* gene. However, a small number of data that was collected over a long time period is a limitation to the reliability of our findings. Therefore, more definitive studies would require data input from much higher number of patients and, preferably, from more than one institution.
